# Joint Transmit–Receive Weight Optimization for FDA Radar to Balance Active Detection and RF Stealth

**DOI:** 10.3390/s26092850

**Published:** 2026-05-02

**Authors:** Haoliang Guan, Shunsheng Zhang, Wen-Qin Wang

**Affiliations:** 1 School of Information and Communication Engineering, University of Electronic Science and Technology of China, Chengdu 611731, China; haoliang@std.uestc.edu.cn (H.G.); wqwang@uestc.edu.cn (W.-Q.W.); 2 Institute of Electronic Science and Technology, University of Electronic Science and Technology of China, Chengdu 611731, China

**Keywords:** frequency diverse array (FDA), radio-frequency (RF) stealth, transmit–receive weight optimization, integrated detection–stealth design

## Abstract

Existing studies on frequency diverse array (FDA) radar sensing systems have primarily focused on radio-frequency (RF) stealth characteristics with limited attention to the balance between RF stealth and active detection performance. To address this issue, this paper proposes a joint transmit–receive weight optimization scheme for FDA radar systems to achieve an effective balance between active detection and RF stealth. The resulting optimization problem is non-convex, and a block coordinate descent (BCD)-based alternating optimization method with a carefully designed initialization strategy is developed to solve it efficiently. Simulation results demonstrate that the proposed method achieves improved RF stealth performance while maintaining comparable active detection capability, compared with conventional FDA radar and representative existing optimization-based benchmark methods. These results demonstrate the effectiveness of the proposed method for balancing active detection and RF stealth performance in FDA radar sensing systems.

## 1. Introduction

The frequency diverse array (FDA) radar, as a novel radar sensing paradigm, has attracted increasing attention in recent years. It was first introduced by Antonik et al. at the U.S. Air Force Research Laboratory [1]. By introducing slight frequency offsets across array elements, FDA radar produces a range–angle–time-dependent transmit beam pattern and provides additional degrees of freedom for sensing-oriented signal design and performance regulation. As a result, FDA radar has been widely investigated in various applications, including target detection [2,3], parameter estimation [4,5], mainlobe jamming suppression [6,7], and radar imaging and sensing [8,9].

In many radar sensing applications, such as airborne or maritime surveillance, the radar system is required not only to actively detect and track targets, but also to reduce the probability of being intercepted by external passive receivers. This requires the radar sensing system to simultaneously maintain reliable active detection performance and low interceptability [10,11]. These requirements make FDA radar particularly attractive for sensing scenarios that require both reliable target sensing and low interceptability [1,2,8].

To the best of the authors’ knowledge, research on the RF stealth performance of FDA radar remains limited. Existing studies in the FDA framework have mainly investigated this topic from two perspectives, namely LPI/RF stealth characteristic analysis and waveform/beamforming design. For example, some studies have examined the LPI and RF-stealth-related characteristics of FDA-transmitted signals and their applications in passive localization deception and counter-DOA estimation [12]. In addition, FDA and FDA-MIMO-related studies have also considered low-probability-of-intercept transmit beamforming, waveform design, and joint transmit waveform–receive beamforming design for improving RF stealth performance [13,14,15]. However, despite these efforts, how to effectively coordinate active detection and RF stealth for FDA radar remains insufficiently explored.

Motivated by this gap, this paper formulates a joint transmit–receive weight optimization problem for FDA radar systems to balance active detection and RF stealth performance. The probability of detection and probability of interception are adopted to characterize the radar-side detection capability and intercept-side exposure risk, respectively. A block coordinate descent (BCD)-based alternating optimization method with a carefully designed initialization strategy is then developed to solve the resulting non-convex optimization problem. In addition, the direction-of-arrival (DOA) estimation accuracy at the intercept receiver is further evaluated in the simulation section to characterize the adversarial parameter estimation capability.

## 2. Signal Model and Problem Formulation

### 2.1. Signal Model

**Figure 1:**
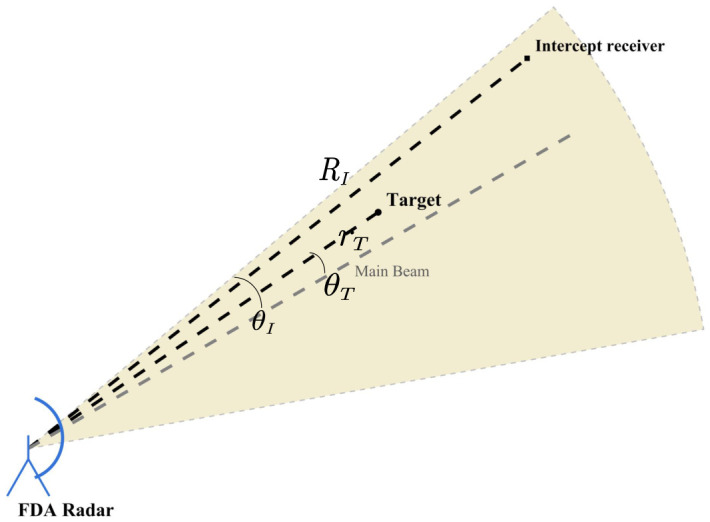
Geometry of the considered FDA radar sensing and interception scenario.

As shown in Figure 1, the considered FDA radar system is used for target sensing, while a potential intercept receiver is located at $\left(R_{I},\theta_{I}\right)$. The FDA radar system is equipped with a uniform linear transmit array of *M* elements and a uniform linear receive array of *N* elements. Let $d_{T}$ and $d_{R}$ denote the inter-element spacings of the transmit and receive arrays, respectively. In the conventional FDA scheme, a fixed frequency increment is applied between adjacent transmit elements. Accordingly, the carrier frequency of the *m*th transmit element is given by

(1) $$f_{m}=f_{0}+m\Delta f,\;m=0,1,\ldots ,M-1$$

where $f_{0}$ is the reference carrier frequency and Δf is the fixed frequency increment. This scheme is referred to as $FDA_{\mathrm{conv}}$ in the simulations.

Let $\varphi_{m}\left(t\right)$ denote the baseband waveform transmitted by the *m*th element over one pulse duration $T_{p}$, and let

(2) $$w_{T}={\left[w_{0},w_{1},\ldots ,w_{M-1}\right]}^{T}\in C^{M\times 1}$$

denote the transmit weight vector. Then, the transmitted signal of the *m*th element can be written as

(3) $$s_{m}\left(t\right)=w_{m}\varphi_{m}\left(t\right)e^{j2\pi f_{m}t},\;0\leq t\leq T_{p}.$$

The transmitted baseband waveforms are assumed to be orthogonal, i.e.,

(4) $$\int_{0}^{T_{p}}\varphi_{m}\left(t\right)\varphi_{m^{\prime }}^{*}\left(t\right)\;dt=\delta_{mm^{\prime }},$$

where $\delta_{mm^{\prime }}$ denotes the Kronecker delta.

For a far-field point target located at (r,θ), the reference round-trip delay is

(5) $$\tau \left(r\right)=\frac{2r}{c},$$

where *c* is the speed of light. The transmit-side and receive-side angular delays are respectively given by

(6) $$\tau_{T,m}\left(\theta \right)=\frac{md_{T}\sin\theta }{c},\;\tau_{R,n}\left(\theta \right)=\frac{nd_{R}\sin\theta }{c},$$

for m=0,1,…,M−1 and n=0,1,…,N−1.

Under the far-field and narrowband assumptions, the echo received at the *n*th receive element due to the *m*th transmit element can be approximated as

(7) $$x_{n,m}\left(t;r,\theta \right)\approx \alpha_{s}w_{m}\varphi_{m}\;\left(t-\tau (r)\right)e^{j2\pi f_{m}\left(t-\tau \left(r\right)\right)}e^{-j2\pi f_{m}\tau_{T,m}\left(\theta \right)}e^{-j2\pi f_{0}\tau_{R,n}\left(\theta \right)},$$

where $\alpha_{s}\in C$ denotes the effective complex scattering coefficient of the target. Define

(8) $$b_{m}\left(r,\theta \right)≜e^{-j2\pi f_{m}\tau \left(r\right)}e^{-j2\pi f_{m}\tau_{T,m}\left(\theta \right)},\;m=0,1,\ldots ,M-1,$$

and stack them into

(9) $$b\left(r,\theta \right)={\left[b_{0}\left(r,\theta \right),b_{1}\left(r,\theta \right),\ldots ,b_{M-1}\left(r,\theta \right)\right]}^{T}\in C^{M\times 1}.$$

Then, the effective transmit vector is

(10) $$u\left(r,\theta \right)=w_{T}⊙b\left(r,\theta \right)\in C^{M\times 1},$$

where ⊙ denotes the Hadamard product.

Under the narrowband receive-array approximation, the receive steering vector is expressed as

(11) $$a_{R}\left(\theta \right)={\left[1,e^{-j2\pi \frac{f_{0}}{c}d_{R}\sin\theta },\ldots ,e^{-j2\pi \frac{f_{0}}{c}\left(N-1\right)d_{R}\sin\theta }\right]}^{T}\in C^{N\times 1}.$$

After multi-channel mixing and matched filtering, ideal channel separation is achieved under the orthogonal waveform assumption, and the output of the (n,m)th channel can be approximated as

(12) $$y_{n,m}\approx \alpha_{s}w_{m}e^{-j2\pi f_{m}\tau \left(r\right)}e^{-j2\pi f_{m}\tau_{T,m}\left(\theta \right)}e^{-j2\pi f_{0}\tau_{R,n}\left(\theta \right)}+\eta_{n,m},$$

where $\eta_{n,m}$ denotes the matched-filter output noise. Stacking all matched-filter outputs yields

(13) $$Y\approx \alpha_{s}\;a_{R}\left(\theta \right)u^{T}\left(r,\theta \right)+N,$$

where $Y,N\in C^{N\times M}$, and $N=[\eta_{n,m}]$ denotes the noise matrix. Let

(14) $$z≜\mathrm{vec}\left(Y\right)\in C^{MN\times 1},\;n≜\mathrm{vec}\left(N\right)\in C^{MN\times 1},$$

where vec(·) denotes the vectorization operator. Then,

(15) $$z\approx \alpha_{s}\left(u\left(r,\theta \right)⊗a_{R}\left(\theta \right)\right)+n,$$

where ⊗ denotes the Kronecker product. The vectorized noise in the matched-filter output domain is assumed to satisfy

(16) $$n∼\mathrm{CN}(0,\sigma_{n}^{2}I_{MN}).$$

based on the above signal model, the receive-side output SNR and the intercept-side SNR are defined in the next subsection for joint optimization. Unlike the radar receiver, which exploits waveform orthogonality through matched filtering, the potential intercept receiver is assumed to observe the superposed transmitted waveform directly without channel separation. Accordingly, the intercept-side SNR is defined based on the average intercepted signal power over one pulse.

### 2.2. Joint Optimization Problem

Consider the desired target located at $\left(r_{T},\theta_{T}\right)$. Define the equivalent target response vector as

(17) $$g_{T}\left(w_{T}\right)≜\left(w_{T}⊙b\left(r_{T},\theta_{T}\right)\right)⊗a_{R}\left(\theta_{T}\right)\in C^{MN\times 1}.$$

Then, the vectorized received signal can be rewritten as

(18) $$z\approx \alpha_{s}\;g_{T}\left(w_{T}\right)+n,$$

and the receive processor output is

(19) $$y=w_{R}^{H}z.$$

Accordingly, the radar-side output SNR is given by

(20) $$\mathrm{SNR}\left(w_{T},w_{R}\right)=\frac{|\alpha_{s}{|}^{2}{\left|w_{R}^{H}g_{T}\left(w_{T}\right)\right|}^{2}}{\sigma_{n}^{2}{∥w_{R}∥}_{2}^{2}}.$$

For an intercept receiver located at $\left(R_{I},\theta_{I}\right)$, the propagation delay is given by $\tau_{I}=R_{I}/c$. Accordingly, the corresponding noiseless intercepted signal is expressed as

(21) $$s_{I}\left(t;\theta_{I},R_{I}\right)=\alpha_{I}\;a_{T}^{T}\left(\theta_{I}\right)\left(\left(\Phi \left(t-\tau_{I}\right)w_{T}\right)⊙e_{I}\left(t-\tau_{I}\right)\right),$$

and the corresponding intercepted signal is

(22) $$y_{I}\left(t;\theta_{I},R_{I}\right)=s_{I}\left(t;\theta_{I},R_{I}\right)+n_{I}\left(t\right),$$

where $\alpha_{I}\in C$ denotes the complex intercepted-signal amplitude, $n_{I}\left(t\right)∼\mathrm{CN}\left(0,\sigma_{I}^{2}\right)$ denotes the intercept-side noise, and

(23) $$a_{T}\left(\theta_{I}\right)={\left[1,\;e^{-j2\pi \frac{f_{0}}{c}d_{T}\sin\theta_{I}},\;\ldots ,\;e^{-j2\pi \frac{f_{0}}{c}\left(M-1\right)d_{T}\sin\theta_{I}}\right]}^{T}\in C^{M\times 1},$$

(24) $$\Phi \left(t\right)=\mathrm{diag}\left\{\varphi_{0}\left(t\right),\varphi_{1}\left(t\right),\ldots ,\varphi_{M-1}\left(t\right)\right\}\in C^{M\times M},$$

and

(25) $$e_{I}\left(t\right)={\left[1,\;e^{j2\pi \Delta ft},\;\ldots ,\;e^{j2\pi (M-1)\Delta ft}\right]}^{T}\in C^{M\times 1}.$$

The average intercepted signal power over one pulse is defined as

(26) $$P_{s,I}\left(w_{T}\right)≜\frac{1}{T_{p}}\int_{0}^{T_{p}}{\left|s_{I}\left(t;\theta_{I},R_{I}\right)\right|}^{2}dt.$$

after expansion, it can be written in quadratic form as

(27) $$P_{s,I}\left(w_{T}\right)={\left|\alpha_{I}\right|}^{2}\;w_{T}^{H}R_{I}w_{T},$$

where

(28) $$R_{I}≜\mathrm{diag}\;{\left(a_{T}\left(\theta_{I}\right)\right)}^{H}R_{\phi }\left(\Delta f,\varphi \right)\mathrm{diag}\;\left(a_{T}\left(\theta_{I}\right)\right)\in C^{M\times M},$$

and the equivalent waveform-coupling matrix is defined by

(29) $${\left[R_{\phi }\left(\Delta f,\varphi \right)\right]}_{m,m^{\prime }}≜\frac{1}{T_{p}}\int_{0}^{T_{p}}\varphi_{m}\left(t\right)\varphi_{m^{\prime }}^{*}\left(t\right)e^{j2\pi (m-m^{\prime })\Delta ft}\;dt,\;m,m^{\prime }=0,1,\ldots ,M-1.$$

Unlike the matched-filter output at the radar receiver, the matrix $R_{\phi }\left(\Delta f,\varphi \right)$ is retained here in its general form and is not approximated by the identity matrix. Therefore, the intercept-side SNR is given by

(30) $${\mathrm{SNR}}_{I}\left(w_{T}\right)=\frac{P_{s,I}\left(w_{T}\right)}{\sigma_{I}^{2}}=\frac{|\alpha_{I}{|}^{2}}{\sigma_{I}^{2}}\;w_{T}^{H}R_{I}w_{T}.$$

The intercept-side SNR provides the basis for evaluating the interception probability and is also related to the adversarial parameter estimation capability after successful interception. In the simulation section, the DOA estimation accuracy at the intercept receiver is further examined as a representative example.

To balance active detection and RF stealth performance, the following weighted objective function is adopted:

(31) $$\Gamma \left(w_{T},w_{R}\right)=\alpha_{1}\frac{{\mathrm{SNR}}_{I,0}}{{\mathrm{SNR}}_{I}\left(w_{T}\right)}+\left(1-\alpha_{1}\right)\frac{\mathrm{SNR}(w_{T},w_{R})}{{\mathrm{SNR}}_{D,0}},\;0\leq \alpha_{1}\leq 1$$

where ${\mathrm{SNR}}_{I,0}={\mathrm{SNR}}_{I}\left(w_{T}^{\left(0\right)}\right)$ and ${\mathrm{SNR}}_{D,0}=\mathrm{SNR}\left(w_{T}^{\left(0\right)},w_{R}^{\left(0\right)}\right)$ are the fixed reference SNRs obtained from the initial feasible FDA configuration and expressed in linear scale, so both terms in (31) become dimensionless and are scaled to a similar order of magnitude. The weighting factor $\alpha_{1}$ controls the trade-off between RF stealth and active detection performance, thereby enabling a flexible balance between the two objectives. Notably, the reciprocal structure of the intercept-side term acts as a stealth-oriented objective component by converting the reduction in the intercept-side SNR into a maximization-oriented design criterion. Compared with a simple linear combination of the radar-side and intercept-side SNRs, this formulation more explicitly captures the opposite design requirements of active detection enhancement and RF stealth improvement.

Accordingly, the joint transmit–receive weight optimization problem is formulated as

(32) $$\begin{array}{cc} \max_{w_{T},w_{R}}\; & \Gamma (w_{T},w_{R})\\ s.t.\; & ∥w_{T}-w_{T0}{∥}_{2}^{2}\leq \varepsilon ,\\ \; & ∥w_{T}{∥}_{2}^{2}=M. \end{array}$$

here, $w_{T0}\in C^{M\times 1}$ denotes the target-oriented reference transmit weight vector, and ε controls the allowable deviation from this reference. Under the transmit-power constraint in (32), the constraint yields the following conservative lower bound on the normalized target-direction power gain η:

(33) $$\varepsilon \leq 2M(1-\sqrt{\eta })$$

thus, ε can be selected according to the allowable target-direction gain loss. In the simulations, M=16 and ε=2 correspond to a worst-case gain loss bounded by approximately 0.56 dB.

Problem (32) is non-convex and generally difficult to solve globally due to the nonlinear objective and the coupling between the transmit and receive weight vectors. Under the considered signal model, active detection performance is mainly preserved by the target-oriented transmit initialization, the similarity constraint, and the receive-side SNR maximization, whereas RF stealth is primarily controlled through transmit weight design. Therefore, an efficient alternating optimization strategy is developed in the next section to obtain a high-quality suboptimal solution.

## 3. Proposed Optimization Method

To solve (32), a block coordinate descent (BCD)-based alternating optimization method is adopted. Specifically, the optimization variables are partitioned into two blocks, namely the transmit weight vector $w_{T}$ and the receive weight vector $w_{R}$. In each iteration, the receive weight vector is updated by maximizing the radar-side output SNR for the current transmit weight vector, followed by a relaxation step for numerical stability, while the transmit weight vector is updated by reducing the intercept-side SNR subject to the similarity and power constraints. The proposed method consists of transmit weight initialization, transmit weight update, receive weight update, and the corresponding alternating optimization algorithm.

### 3.1. Transmit Weight Initialization

Since the performance of the BCD algorithm is sensitive to initialization, a target-oriented reference transmit weight vector is first constructed. Specifically, before introducing the RF stealth requirement into the transmit weight design, the transmit weight vector is initialized by maximizing the coherent transmit gain toward the desired target location $\left(r_{T},\theta_{T}\right)$ under a fixed transmit-power constraint. Let

(34) $$b_{T}≜b\left(r_{T},\theta_{T}\right)\in C^{M\times 1},$$

where b(r,θ) is defined in the previous subsection. The reference transmit weight vector is obtained by solving

(35) $$\begin{array}{cc} \max_{w_{T}}\; & {\left|b_{T}^{T}w_{T}\right|}^{2}\\ s.t.\;\; & ∥w_{T}{∥}_{2}^{2}=M. \end{array}$$

According to the Cauchy–Schwarz inequality, the optimal solution to (35) is given by

(36) $$w_{T0}=\sqrt{M}\;\frac{b_{T}^{*}}{∥b_{T}{∥}_{2}}.$$

it is straightforward to verify that $∥w_{T0}{∥}_{2}^{2}=M$. The obtained $w_{T0}$ is then used as the reference transmit weight vector in the subsequent similarity constraint.

### 3.2. Transmit Weight Update

Under the alternating optimization framework, the transmit weight vector is refined to reduce the intercept-side SNR while preserving the target-oriented transmit characteristic through the similarity constraint. Accordingly, the transmit weight update is carried out by solving

(37) $$\begin{array}{cc} \min_{w_{T}}\; & {\mathrm{SNR}}_{I}\left(w_{T}\right)=\frac{|\alpha_{I}{|}^{2}}{\sigma_{I}^{2}}w_{T}^{H}R_{I}w_{T}\\ s.t.\; & ∥w_{T}-w_{T0}{∥}_{2}^{2}\leq \varepsilon ,\\ \; & ∥w_{T}{∥}_{2}^{2}=M. \end{array}$$

The gradient of ${\mathrm{SNR}}_{I}\left(w_{T}\right)$ with respect to $w_{T}^{*}$ is given by

(38) $$\nabla_{w_{T}^{*}}{\mathrm{SNR}}_{I}=\frac{|\alpha_{I}{|}^{2}}{\sigma_{I}^{2}}R_{I}w_{T}.$$

the descent direction at the *k*th iteration is defined as

(39) $$d_{T}^{\left(k\right)}=\nabla_{w_{T}^{*}}{\mathrm{SNR}}_{I}\left(w_{T}^{\left(k\right)}\right).$$

For a candidate step size μ>0, construct the normalized trial point as

(40) $${\tilde{w}}_{T}\left(\mu \right)=\sqrt{M}\;\frac{w_{T}^{\left(k\right)}-\mu d_{T}^{\left(k\right)}}{∥w_{T}^{\left(k\right)}-\mu d_{T}^{\left(k\right)}{∥}_{2}},$$

which automatically satisfies the power constraint. The step size is selected by backtracking line search. Specifically, let $\mu =\mu_{0}\beta^{\ell }$, where $\mu_{0}>0$, 0<β<1, and ℓ=0,1,2,…. Starting from ℓ=0, the smallest *ℓ* is chosen such that

(41) $$∥{\tilde{w}}_{T}\left(\mu \right)-w_{T0}{∥}_{2}^{2}\leq \varepsilon$$

and

(42) $${\mathrm{SNR}}_{I}\left({\tilde{w}}_{T}\left(\mu \right)\right)\leq {\mathrm{SNR}}_{I}\left(w_{T}^{\left(k\right)}\right).$$

The transmit weight vector is then updated as

(43) $$w_{T}^{\left(k+1\right)}={\tilde{w}}_{T}\left(\mu_{k}\right),$$

where $\mu_{k}$ denotes the accepted step size at the *k*th iteration.

### 3.3. Receive Weight Update

For a fixed transmit weight vector $w_{T}^{\left(k\right)}$, define

(44) $$g_{T}^{\left(k\right)}≜g_{T}\left(w_{T}^{\left(k\right)}\right)=\left(w_{T}^{\left(k\right)}⊙b\left(r_{T},\theta_{T}\right)\right)⊗a_{R}\left(\theta_{T}\right).$$

Then, the receive weight update is obtained by maximizing the radar-side output SNR, namely

(45) $$\max_{w_{R}}\;\frac{{\left|w_{R}^{H}g_{T}^{\left(k\right)}\right|}^{2}}{∥w_{R}{∥}_{2}^{2}}.$$

Under the white noise assumption, (45) is a standard Rayleigh quotient problem. Equivalently, it can be viewed as a special case of the minimum variance distortionless response (MVDR) filter when the interference-plus-noise covariance matrix reduces to a scaled identity matrix. Therefore, the intermediate receive weight vector is first obtained as

(46) $${\hat{w}}_{R}^{\left(k+1\right)}=\frac{g_{T}^{\left(k\right)}}{∥g_{T}^{\left(k\right)}{∥}_{2}}.$$

To improve numerical stability and avoid abrupt receiver adaptation during the iterative process, a relaxation step is further introduced:

(47) $$w_{R}^{\left(k+1\right)}=\frac{\left(1-\rho \right)w_{R}^{\left(k\right)}+\rho {\hat{w}}_{R}^{\left(k+1\right)}}{{∥\left(1-\rho \right)w_{R}^{\left(k\right)}+\rho {\hat{w}}_{R}^{\left(k+1\right)}∥}_{2}},\;0<\rho \leq 1,$$

where ρ is the relaxation factor.

### 3.4. BCD-Based Alternating Optimization Algorithm

Based on the above two update steps, the overall alternating optimization algorithm is summarized as follows.

1. Initialize the transmit weight vector as $w_{T}^{\left(0\right)}=w_{T0}$ using (36), and initialize the receive weight vector as
  (48) $$w_{R}^{\left(0\right)}=\frac{g_{T}\left(w_{T}^{\left(0\right)}\right)}{∥g_{T}\left(w_{T}^{\left(0\right)}\right){∥}_{2}}.$$
  Set the relaxation factor ρ, the maximum iteration number $K_{\max}$, and the convergence threshold ξ, and let k=0.
2. For the current transmit weight vector $w_{T}^{\left(k\right)}$, compute the intermediate receive weight vector ${\hat{w}}_{R}^{\left(k+1\right)}$ according to (46), and then update $w_{R}^{\left(k+1\right)}$ according to (47).
3. Update the transmit weight vector $w_{T}^{\left(k+1\right)}$ according to (38)–(43).
4. Evaluate the relative change in the intercept-side SNR. If
  (49) $$\frac{\left|{\mathrm{SNR}}_{I}\left(w_{T}^{\left(k+1\right)}\right)-{\mathrm{SNR}}_{I}\left(w_{T}^{\left(k\right)}\right)\right|}{\left|{\mathrm{SNR}}_{I}\left(w_{T}^{\left(k\right)}\right)\right|}\leq \xi$$
  or $k\geq K_{\max}$, terminate the iteration; otherwise, set k←k+1 and repeat Steps 2–4.

The above iterative procedure yields a high-quality suboptimal solution that reduces the intercept-side SNR while preserving active detection capability through target-oriented transmit initialization, similarity-constrained transmit updating, and relaxation-enhanced receive-side SNR maximization.

### 3.5. Computational Complexity and Hardware Feasibility Analysis

Assuming that the iteration-independent quantities have been precomputed, the computational complexity of the proposed BCD algorithm is analyzed as follows. Here, *K* is the number of outer iterations, and $L_{\mathrm{ls}}$ is the average number of backtracking line-search trials per transmit weight update. For the receive weight update, the main cost comes from constructing the equivalent target response vector $g_{T}^{\left(k\right)}$, whose dimension is MN×1. Thus, the complexity of this step is O(MN). For the transmit weight update, the main cost comes from gradient evaluation and repeated objective-function evaluations during backtracking line search, which are dominated by operations over the *M*-dimensional transmit weight vector. Therefore, the complexity of this step is $O(L_{\mathrm{ls}}M^{2})$. Consequently, the computational complexity per outer iteration is $O(MN+L_{\mathrm{ls}}M^{2})$, and the total complexity after *K* iterations is $O\left(K(MN+L_{\mathrm{ls}}M^{2})\right)$.

During radar operation, the proposed optimization is performed over a dwell or update interval using coarse spatial information extracted from previous echoes. The updated $w_{R}$ and $w_{T}$ are then applied to receive processing and subsequent pulse transmission, respectively. Since the online computation mainly consists of matrix–vector multiplications, vector construction, normalization, projection, and backtracking line search, without semidefinite programming, eigenvalue decomposition, or large-scale matrix inversion, the algorithm is suitable for parallel implementation on SDR-, FPGA-, or RFSoC-based radar platforms, such as the NI Ettus USRP X410 and AMD ZCU111 RFSoC evaluation kit [16,17].

## 4. Simulation Results

In this section, the proposed method is evaluated through numerical simulations and measured-data validation. The simulations assess the detection and stealth balance, whereas the measured-data results verify the active detection capability using real radar echoes.In this section, the proposed method is evaluated through numerical simulations and measured-data validation. The simulations assess the detection and stealth balance, whereas the measured-data results verify the active detection capability using real radar echoes. The main simulation parameters are summarized in Table 1.

### 4.1. Joint Simulation Results of Active Detection and RF Stealth

**Figure 2:**
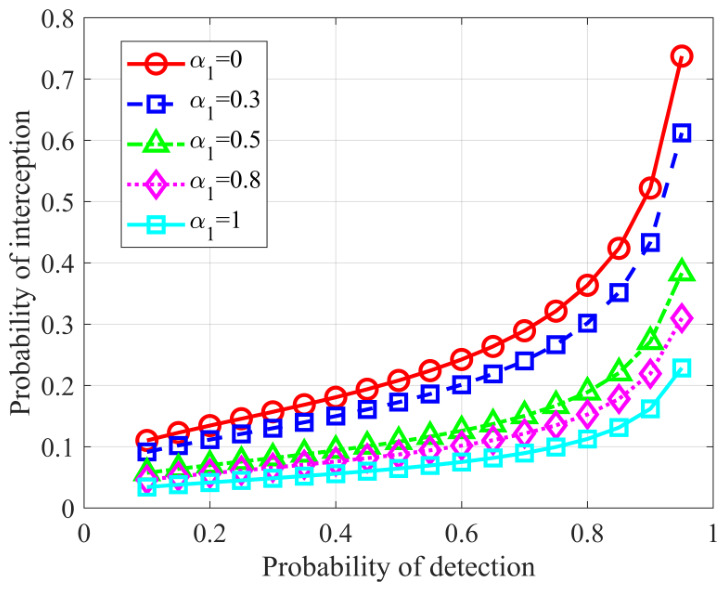
Balance between active detection performance and RF stealth performance under different weighting factors.

We first evaluate the detection and stealth balance achieved by the proposed method. Figure 2 shows the probability of detection versus the probability of interception under different values of the weighting factor $\alpha_{1}$. As $\alpha_{1}$ increases, the optimization places greater emphasis on RF stealth, resulting in a lower interception probability with a slight reduction in detection probability. These results indicate that $\alpha_{1}$ provides an effective mechanism for adjusting the balance between active detection and RF stealth according to different operational requirements.

**Figure 3:**
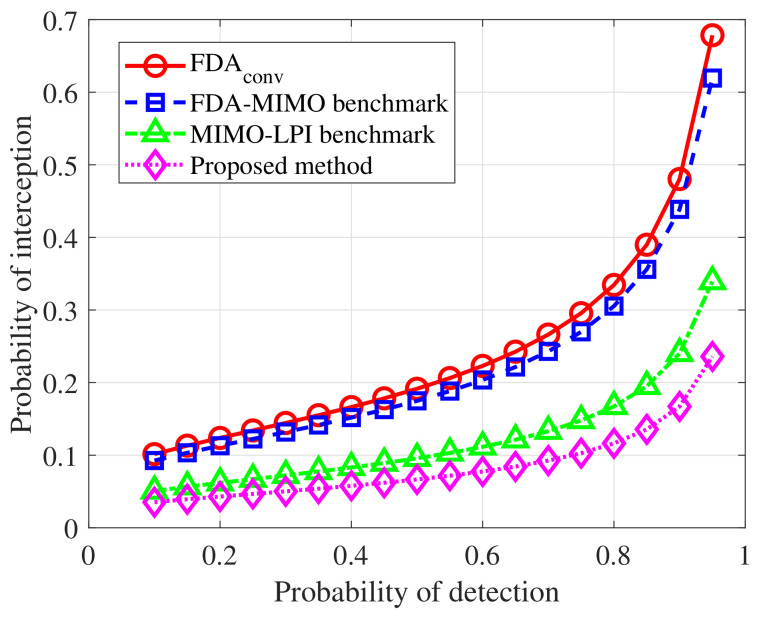
Detection and interception performance comparison under the RF-stealth-emphasized setting. The FDA-MIMO benchmark and MIMO-LPI benchmark correspond to the methods in [15] and [18], respectively.

Figure 3 compares the detection and stealth balance of different radar schemes under the RF-stealth-emphasized setting, i.e., $\alpha_{1}=0.8$. For comparison, the methods in [15,18] are selected as two representative benchmarks, corresponding to FDA-MIMO low-intercept transmit beampattern optimization and transmit–receive joint LPI beamforming under a transmit-subaperturing MIMO architecture, respectively. Compared with the conventional FDA scheme and the two benchmark methods, the proposed method achieves a more favorable balance between active detection and RF stealth. Specifically, for a given detection probability, the proposed method yields a lower probability of interception, especially in the high-detection-probability region. These results demonstrate the effectiveness of the proposed transmit–receive optimization strategy in balancing active detection capability and RF stealth performance.

**Figure 4:**
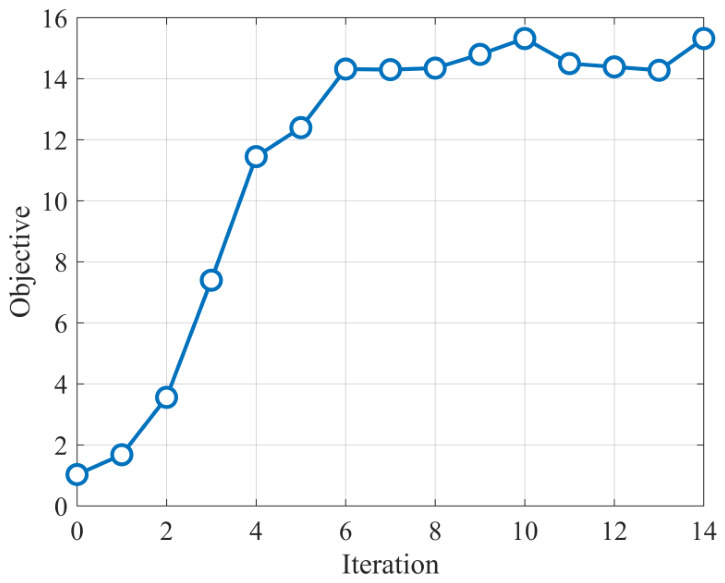
Convergence behavior of the proposed transmit–receive optimization algorithm under $\alpha_{1}=0.8$.

To further verify the stability of the proposed optimization procedure, Figure 4 shows the convergence behavior of the proposed transmit–receive optimization algorithm under $\alpha_{1}=0.8$. It can be observed that the objective value decreases rapidly during the first several iterations and then gradually approaches a stable value. This result indicates that the proposed alternating optimization algorithm can achieve stable convergence within a limited number of iterations, thereby supporting the effectiveness and practical applicability of the proposed method.

**Figure 5:**
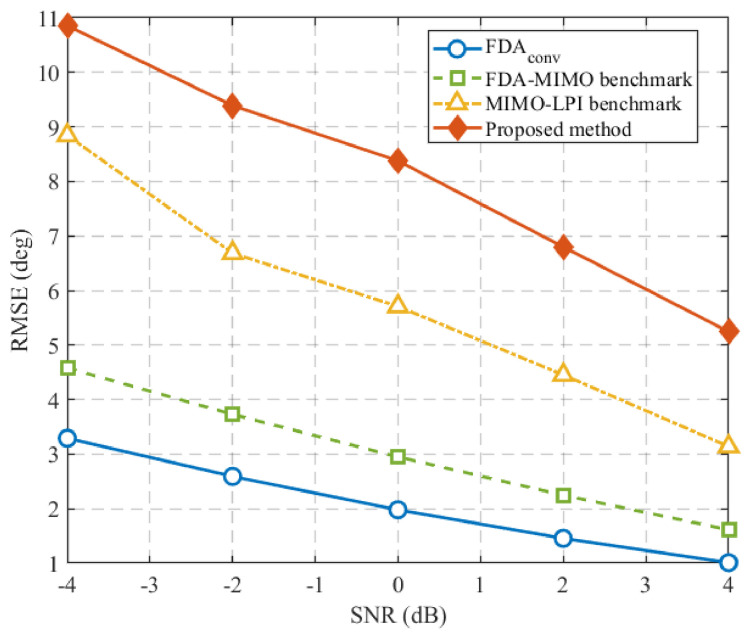
RF stealth performance comparison in terms of DOA estimation RMSE at the intercept receiver. The FDA-MIMO benchmark and MIMO-LPI benchmark correspond to the methods in [15] and [18], respectively.

Figure 5 compares the RF stealth performance of different radar schemes in terms of the DOA estimation RMSE at the intercept receiver. As the intercept-side SNR increases, the RMSE of all schemes decreases due to the improved observation quality at the intercept receiver. However, the proposed method consistently yields the largest RMSE over the considered SNR range, indicating that it can more effectively degrade the DOA estimation capability of the intercept receiver. This result further verifies the RF stealth advantage of the proposed method from the perspective of adversarial DOA estimation.

### 4.2. Complexity and Run-Time Analysis

To further evaluate the computational efficiency of the proposed algorithm, the theoretical complexity and the measured run time are analyzed in this subsection.

Table 2 summarizes the per-iteration computational complexity of different methods. The SDP-based method generally suffers from high computational complexity due to the use of an interior-point solver. The method in [18] involves matrix inversion or equivalent joint beamforming updates, resulting in a cubic-order computational burden. In contrast, the proposed method mainly relies on matrix–vector multiplications and a simple line-search procedure, whose complexity is approximately $O(L_{\mathrm{ls}}M^{2}+MN)$. Therefore, the proposed method can substantially reduce the computational burden.

To evaluate the practical run-time performance, all methods are implemented under the same computational environment. The simulations are carried out in MATLAB R2022a on a computer equipped with an Intel Core i7-9700K CPU and 24 GB RAM. No GPU acceleration or parallel computing is used.

**Figure 6:**
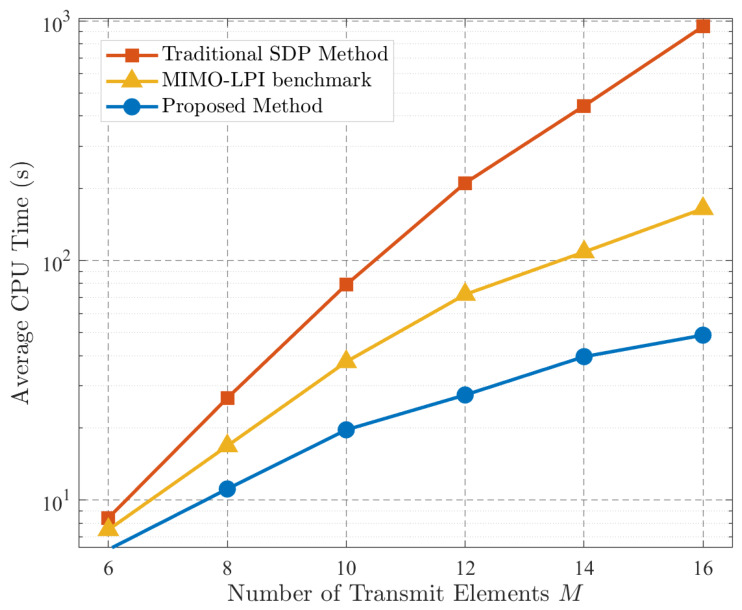
Average CPU time comparison versus the number of transmit elements. The MIMO-LPI benchmark corresponds to the method in [18].

Figure 6 shows the CPU time of the traditional SDP method, the MIMO-LPI benchmark in [18], and the proposed method as the number of transmit elements increases. It can be observed that the proposed method exhibits a slower growth in runtime than the baseline methods. This result is consistent with the theoretical complexity analysis in Table 2, further confirming the computational efficiency of the proposed algorithm. Hence, the proposed method has better potential for real-time implementation in practical FDA radar systems.

### 4.3. Measured-Data Validation of Active Detection Performance

To further validate the active detection performance, measured data from the publicly available LSS-DAUR-1.0 dataset [19] were used. Specifically, the target Doppler complex data of a fixed-wing UAV were selected, and the corresponding time–Doppler response was used for evaluation.

**Figure 7:**
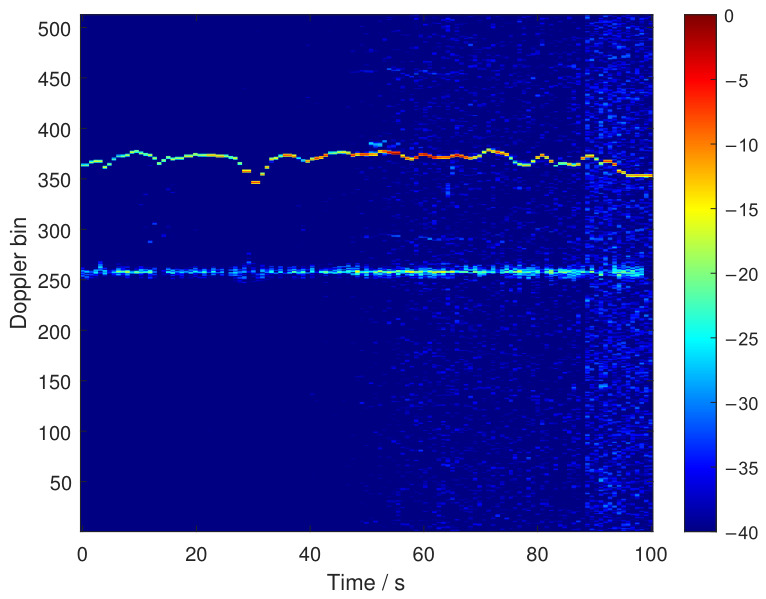
Time–Doppler response obtained from the measured TD data of a fixed-wing UAV for active detection validation.

Figure 7 shows the time–Doppler response obtained from the measured TD data of the fixed-wing UAV. A distinct energy ridge can be observed in the nonzero Doppler region, indicating that the target echo exhibits a stable Doppler signature during the observation interval. This result is consistent with the associated track information and confirms that the adopted measured data contain reliable target motion information. Therefore, the measured-data resultprovides supplementary validation of the active detection performance considered in this work.

It should be noted that the measured-data validation in this subsection is intended to provide supplementary evidence for the active detection performance. The RF stealth performance is evaluated based on the proposed intercept-side model and the corresponding numerical simulations.

## 5. Conclusions

In this paper, a joint transmit–receive weight optimization scheme was developed for FDA radar to balance active detection and RF stealth performance. Simulation results show that the proposed method achieves a lower probability of interception than the conventional FDA scheme and the benchmark methods while maintaining comparable detection performance. The intercept-receiver DOA estimation results and run-time analysis further demonstrate its RF stealth advantage and computational feasibility. Overall, the proposed scheme provides an effective design approach for improving RF stealth capability in FDA radar systems.

## Figures and Tables

**Table 1:** Simulation parameters.

Parameter	Symbol	Value
Number of transmit elements	*M*	16
Number of receive elements	*N*	16
Frequency offset	Δf	2
Carrier frequency	$f_{0}$	5
Target slant range	$r_{T}$	5
Target azimuth angle	$\theta_{T}$	0
Number of integrated pulses	$N_{p}$	16
False alarm probability	$p_{fa}$	$10^{-6}$
Intercept receiver azimuth angle	$\theta_{I}$	5
Intercept receiver slant range	$R_{I}$	10
Milaritydeviation	ε	2
Relaxation factor	ρ	0.6
Maximum iteration number	$K_{max}$	50
Convergence threshold	ξ	$10^{-4}$

**Table 2:** Computational complexity comparison of different methods.

Algorithm	Core Computational Bottleneck	Per-Iteration Complexity
SDP-based method	SDP solver	$O(M^{3.5})$–$O(M^{4.5})$
Method in [18]	Matrix inversion/joint beamforming update	$O(M^{3}+N^{3})$
Proposed method	Matrix-vector multiplication and line search	$O(L_{\mathrm{ls}}M^{2}+MN)$

**Note:** *M* and *N* denote the number of transmit and receive elements, respectively, and $L_{\mathrm{ls}}$ is the number of line-search steps in each iteration.

## Data Availability

The measured data used in this study are from the publicly available LSS-DAUR-1.0 dataset. The simulation data generated in this study are available from the corresponding author upon reasonable request.
